# Somatic mosaicism by a de novo* MLH1* mutation as a cause of Lynch syndrome

**DOI:** 10.1002/mgg3.699

**Published:** 2019-05-18

**Authors:** Willemina R. Geurts‐Giele, Efraim H. Rosenberg, Anja van Rens, Monique E. van Leerdam, Winand N. Dinjens, Fonnet E. Bleeker

**Affiliations:** ^1^ Department of Pathology Erasmus MC Cancer Institute, University Medical Center Rotterdam the Netherlands; ^2^ Department of Pathology Netherlands Cancer Institute Amsterdam the Netherlands; ^3^ Familial Cancer Clinic, Netherlands Cancer Institute Amsterdam the Netherlands; ^4^ Department of Gastroenterology Netherlands Cancer Institute Amsterdam the Netherlands

**Keywords:** Lynch syndrome, MLH1, mosaicism

## Abstract

**Background:**

Lynch syndrome (LS) is caused by germline mismatch repair (MMR) gene mutations. De novo MMR gene mutations are rare, and somatic mosaicism in LS is thought to be infrequent. We describe the first case of somatic mosaicism by a de novo* MLH1* mutation for a patient diagnosed with a rectosigmoid adenocarcinoma at age 31.

**Methods:**

Twelve years after initial colorectal cancer diagnosis, tumor tissue of the patient was tested with sensitive next generation sequencing (NGS) analysis for the presence of somatic MMR mutations.

**Results:**

In tumor tissue, an inactivating *MLH1* mutation (c.518_519del; p.(Tyr173Trpfs*18)) was detected, which was also present at low level in the blood of the patient. In both parents, as well as the patient's sisters, the mutation was not present.

**Conclusion:**

We show that low‐level mosaicism can be detected by using high‐coverage targeted NGS panels on constitutional and/or tumor DNA. This report illustrates that by using sensitive sequencing techniques, more cases of genetic diseases driven by mosaic mutations may be identified, with important clinical consequences for patients and family members.

## 1 INTRODUCTION

Heterozygous germline mutations in MMR genes cause Lynch syndrome (LS), an autosomal dominant condition which predisposes to various types of cancer including colorectal cancer (CRC) and endometrial cancer (Hampel et al., 2008, 2006). In contrast to other hereditary CRC syndromes, for example Familial Adenomatous Polyposis, de novo germline mutations in MMR genes are described (Plasilova et al., 2006; Stulp et al., 2006) but appear to be rare. Only 2.3% of MMR gene mutation carriers are reported to have a de novo mutation (Win et al., 2011). Biallelic somatic MMR gene aberrations have been described more frequently, and account for 50%–70% of microsatellite instable (MSI) tumors without causal germline mutations or promotor methylation (Geurts‐Giele et al., 2014; Haraldsdottir et al., 2014; Mensenkamp et al., 2014). Somatic mosaicism is the presence of two or more populations of cells with different genotypes in one individual, and can cause the development of neoplasia if involving a gene related with oncogenesis (Hall, 1988). Somatic mosaicism in LS is thought to be very infrequent; to our knowledge, only two cases have been described thus far. Pastrello et al. (2009) describe somatic mosaicism in a LS patient caused by reversion of an inherited mutation in *MLH1* (OMIM 120436), this mutation was present in >80% of both blood and tissues. Furthermore, Sourrouille et al. (2013) describe a patient with somatic mosaicism due to a de novo mutation in *MSH2* (OMIM 609309), which was detected in normal colonic tissue but not in the blood. This mosaic *MSH2* mutation apparently had germline transmission since this mutation was also present in the patient's son. Here, we report the first case of somatic mosaicism by a de novo* MLH1* mutation.

Our male index patient was diagnosed with a rectosigmoid adenocarcinoma at the age of 31. The patient had no relevant medical history. He has two healthy sisters and no offspring. Both his father and mother did not develop any tumor during life and died at 85 and 80 years of age, respectively. A brother of his mother had bladder cancer at age 67, and grandfather from mother's side died from liver cancer diagnosed at age 66.

Because of his young age, he was referred for genetic counseling to the Familial Cancer Clinic. Immunohistochemistry (IHC) of the MMR proteins was performed on the tumor tissue, which showed normal nuclear expression of MSH2 and MSH6 but absence of nuclear expression of MLH1 and PMS2. Molecular analyses showed MSI without *MLH1* promotor methylation in the tumor tissue. Mutation scanning of the four MMR genes by denaturing gradient gel electrophoresis (DGGE) of DNA isolated from blood showed no indication for the presence of a germline mutation. The bladder tumor of the index patient's uncle was also examined but did not show any signs of MSI (IHC was not possible). Based on these results, the patient was considered to be Lynch‐like. Therefore he and his sisters underwent colonoscopy every 2 years, no polyps were identified. Additionally, surveillance for extra‐colonic tumors was advised to him and his sisters. One sister decided to have her ovaries and uterus preventively removed at 46 years of age. In both organs, no signs of neoplastic growth were observed.

Recently, all Lynch‐like patients of the Familial Cancer Clinic at the Netherlands Cancer Institute are being evaluated for somatic MMR gene mutations, as the presence of a somatic MMR gene mutation can indicate a sporadic origin of the tumor. Therefore, last year the index patient visited the Familial Cancer Clinic again, 12 years after the initial CRC diagnosis. Subsequently, the tumor was tested with next generation sequencing (NGS) for the presence of somatic MMR mutations.

## 2 MATERIALS AND METHODS

### 2.1 Ethical compliance

The index patient was counseled by the Familial Cancer Clinic and provided informed consent.

### 2.2 Somatic mutation analysis of the MMR genes

Normal and tumor tissues of the index patient were manually microdissected from five to ten hematoxylin‐stained sections of formalin‐fixed paraffin‐embedded (FFPE) tissue, after which proteinase K and 5% Chelex 100 resin was added for DNA isolation, as previously described (van Lier et al., 2010). DNA concentrations were measured with the Qubit 2.0 Fluorometer and 10 ng DNA input was used for mutation analysis. Mutation analysis with the Ion S5 XL system (Ion Torrent) was performed with suppliers' materials and protocols (Thermo Fisher Scientific). A custom‐made primer panel was designed using the ampliseq designer. This panel targets the open reading frame including the intron–exon boundaries of *MLH1* (NM_000249.3), *MSH2* (NM_000251.2), *MSH6* (OMIM 600678, NM_000179.2), and *PMS2* (OMIM 600259, NM_000535.7), with a coding sequence coverage of 97%, 94%, 95%, and 81% respectively, and hotspots for *BRAF* (OMIM 164757, NM_004333.5; exon 11 and 15), *POLD1* (OMIM 174761, NM_002691.3; exon 12), and *POLE* (OMIM 174762, NM_006231.3; exon 3 and 13). Furthermore, single nucleotide polymorphisms in and around the *MLH1*, *MSH2*, *MSH6*, and *PMS2* genes are included to detect copy number variations, as previously described (Dubbink et al., 2016). Primer sequences are available on request.

Libraries were made using the Ion AmpliSeq Library Kit 2.0‐384 LV according to the Ion Ampliseq Library Preparation User Guide. Template was performed with the Ion 510/520/530 Chef kit and sequencing was performed on a 530 chip using the Ion S5 XL system. Data were analyzed using SeqPilot version 4.2.2 (JSI medical systems).

### 2.3 Sanger sequencing of MLH1

DNA isolation from blood of the index patient and his two sisters was performed using DNAzol (Thermo Fischer Scientific). DNA from normal FFPE tissue of the deceased parents was isolated using QIA Amp DNA purification kit (Qiagen). Sanger sequencing for *MLH1* exon 6 was performed using standard procedures.

## 3 RESULTS

**Figure 1:**
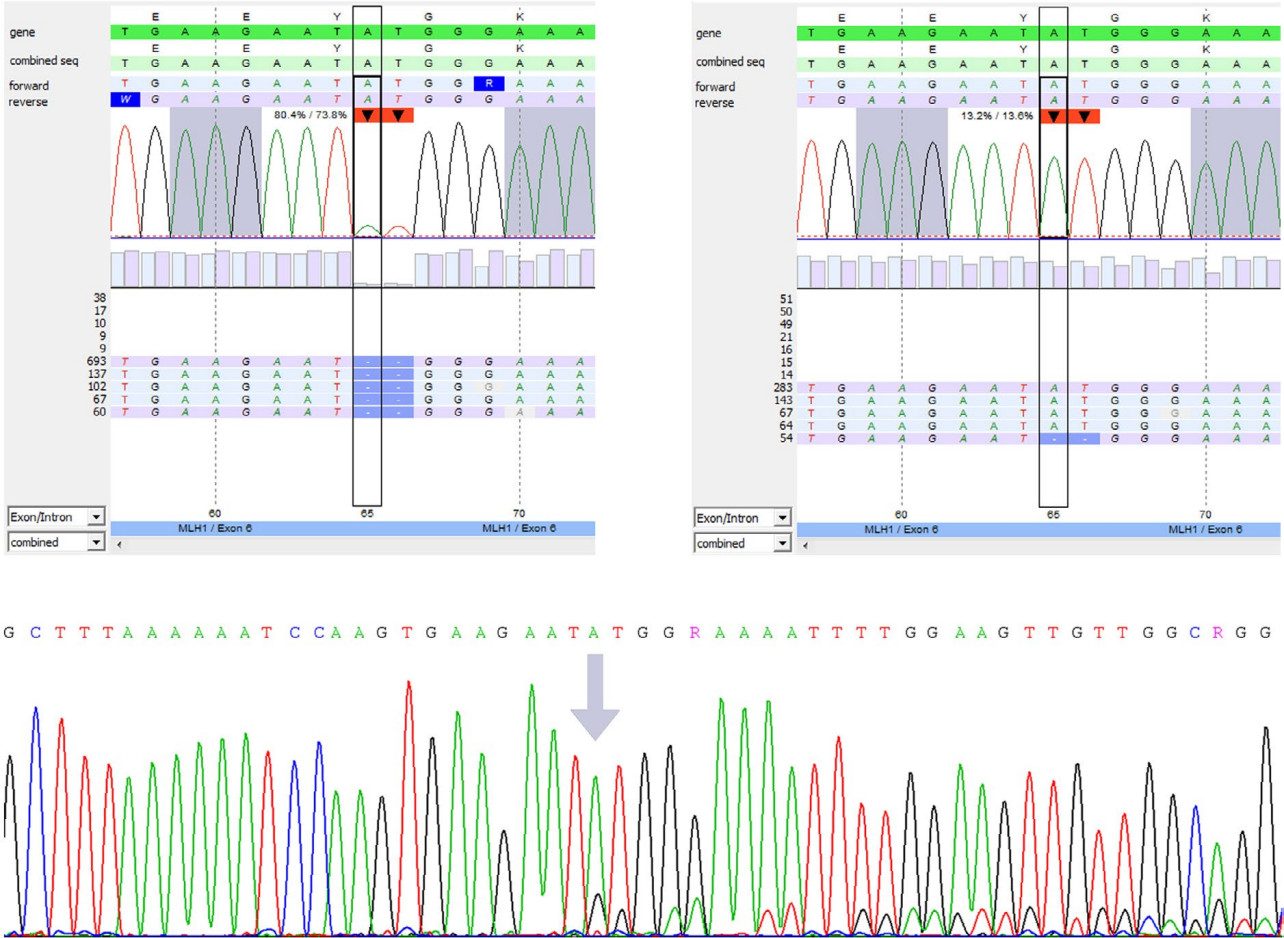
*MLH1*ª c.518_519del; p.(Tyr173Trpfs*18) analysis. (a) NGS analysis of the colon adenocarcinoma showing the mutation in 80% of the reads. (b) NGS analysis of DNA isolated from blood showing the mutation in 13% of the reads. (c) Sanger sequencing of DNA isolated from blood confirming the presence of the *MLH1* mutation (arrow indicates start of the deletion). ªOMIM 120436, NM_000249.3. NGS, next generation sequencing

In the tumor, an inactivating *MLH1* mutation (c.518_519del; p.(Tyr173Trpfs*18); see Figure 1) was identified and loss of the wild‐type *MLH1* allele was seen, indicating biallelic *MLH1* inactivation. This explains the MSI phenotype and the absence of nuclear expression of MLH1 and PMS2 in the tumor cells. Additionally, five missense variants of unknown significance were detected in tumor tissue only, one in *MSH2* (c.2198C>A; p.(Ala733Asp)) and four in *MSH6 (*c.821G>A; p.(Ser274Asn), c.1730G>A; p.(Arg577His), c.2419G>A; p.(Glu807Lys) and c.3163G>A; p.(Ala1055Thr))*.*

Surprisingly, the *MLH1* mutation was also detected by NGS at low frequencies in DNA isolated from several normal tissues, including smooth muscle near the tumor, epithelium from the small intestine and lymph nodes without metastasis (see Table 1). NGS showed the presence of the inactivating *MLH1* mutation with a variant allele frequency of 13% in the blood sample, a level that would not generally be detectable by DGGE. These results suggested mosaicism of the *MLH1* mutation in at least two germ layers. To rule out reversion of an inherited *MLH1* mutation as described before (Pastrello et al., 2009), normal tissue of the deceased parents was tested using Sanger sequencing for the presence of the *MLH1* mutation. In both parents, the mutation was not detected, indicating the appearance of a post‐zygotic de novo mutation in the index patient. As expected, the mutation was also not identified in the DNA isolated from blood of the patient's two sisters. Therefore his sisters were dismissed from further LS surveillance.

**Table 1:** Different tissues from the rectosigmoid resection of the index patient (including part of the bladder and ileum), as well as DNA isolated from blood, were included for somatic MMR gene sequencing analysis

Tissue	Neoplastic cell content as estimated by a GE pathologist	Variant allele frequency (VAF) of *MLH1* a c.518_519del; p.(Tyr173Trpfs*18)
Colon adenocarcinoma	70%	80%^b^
Colon muscle near adenocarcinoma	0%	14%
Small intestine muscle	0%	16%
Small intestine epithelium	0%	14%
Lymph node	0%	14%
Blood sample (in duplo)	Not applicable	13%

Abbreviation: MMR, mismatch repair.

a OMIM 120436, NM_000249.3.

b VAF indicative for loss of the wild‐type allele.

It is thought that somatic mosaicism gives a milder phenotype than full LS depending on the extend of the mosaicism in the cell types prone for malignant transformation (Hall, 1988). However, as the degree of mosaicism can vary strongly, even within an organ or cell type (Forsberg, Gisselsson, & Dumanski, 2017), it is impossible to estimate the patients' risk for various types of cancer based on the mutation rate. Therefore, the index patient is now considered to be a LS patient and is advised the regular LS surveillance. As the patient does not have offspring, we cannot check for gonadal mosaicism. After the above results were obtained, the patient continued the LS surveillance program.

## 4 DISCUSSION

As described previously for other mosaic cases, this report highlights that, as former gene scanning techniques in clinical genetics laboratories will be replaced by deep sequencing techniques, more cases of genetic diseases driven by mosaic mutations may be identified, with important clinical consequences for patients and family members. If no pathogenic mutation is detected in the blood, mutational analysis of tumor tissue might help to identify low‐level mosaicism. All neoplastic cells will most likely harbor the pathogenic mutation, and loss of the wild‐type allele, a common event for tumor suppressor genes, enriches for the pathogenic mutation even further. If a mutation is detected in tumor tissue, the surrounding normal tissue and constitutional DNA can be screened for the presence of this specific mutation with very sensitive techniques (like NGS), enabling detection of low‐frequency mutations.

When routine mutation analysis of constitutional DNA from patients with a clear clinical suspicion for a tumor syndrome is negative, one should consider to perform high‐coverage targeted NGS of the indicated genes to detect possible mosaicism. Furthermore, sequencing of tumor tissue can guide detection of a low‐level mosaic variant in the germline. This diagnostic strategy will result in improved health care both for the index patient and his or her family members.

## CONFLICT OF INTEREST

The authors declare no conflict of interests.
